# Toward a More Conclusive Understanding of the Relationship between Musical Training and Reading

**DOI:** 10.3389/fpsyg.2017.00263

**Published:** 2017-02-27

**Authors:** McNeel G. Jantzen

**Affiliations:** Language and Neural Systems Laboratory, Department of Psychology and Behavioral Neuroscience Program, Western Washington UniversityBellingham, WA, USA

**Keywords:** musical training, language, speech, music, auditory perception, neural plasticity, development, neural overlap

A burgeoning question in understanding literacy development is whether engaging in musical training directly transfers benefits to reading-related skills. Although, there is considerable data examining transfer effects of musical training on literacy skills, results from these studies have been largely inconclusive (Anvari et al., 2002; Overy, 2003; Dellatolas et al., 2009; Melby-Lervåg et al., 2012; Zuk et al., 2013a; Woodruff Carr et al., 2014). To further delineate the potential effects that musical training has on developing reading skills Gordon et al. (2015) reviewed controlled training studies and evaluated whether the research demonstrated positive evidence for a direct transfer hypothesis. Using a meta-analytic approach to evaluate the efficacy of musical training for language outcomes and identify the attributes of music training paradigms that are relevant to specific reading outcomes, the authors focused on literature that addressed training, pre- post-assessment designs, and constancy of reading instruction. This work is an important touchstone for future work for two reasons. First, it highlights with greater specificity what skills may be transferred between musical training and reading. Second, it promotes further investigation of the impact of musical training on literacy skills by outlining potential moderators and study design factors central for future longitudinal work.

Initial results from Gordon et al.'s (2015) meta-analysis show that broad category Phonological Awareness outcomes were slightly greater for children who received musical training vs. control groups. Their results reaffirm existing findings demonstrating that musical training and aptitude may enhance language skills. Notably, musicians show improved phonological awareness skills compared to their non-musician counterparts (Forgeard et al., 2008; Zuk et al., 2013b); music aptitude correlates with phonological skills in children (Lamb and Gregory, 1993; Anvari et al., 2002; Peynircioglu et al., 2002; Dellatolas et al., 2009; Chobert et al., 2011; Tierney and Kraus, 2013), and the positive effects of intensive musical training on phonological representations in dyslexics and normal reading children (Chobert et al., 2014; Habib et al., 2016). However, Gordon and colleagues distinguish their work from previous studies by creating subcategories for Rhyming and Other Phonological outcomes that allowed them to more clearly identify the reading-related skills that may benefit from musical training. Gordon et al.'s approach revealed that 40 h or more of musical training specifically improved rhyming skills. Although, they are cautious to not overstate their findings, Gordon et al.'s (2015) work is consistent with previous research connecting rhythm and reading skills (Dellatolas et al., 2009; Strait and Kraus, 2011; Tierney and Kraus, 2013; Woodruff Carr et al., 2014). In addition to arguing in favor of applying more refined measures of reading-related skills, the authors further suggest that our understanding of the relationship between musical training and reading may benefit from studying a broader range of musical experience beyond formal musical training. For example, similar meta-analyses demonstrate positive results for reading education programs that incorporate musical components into the curricula (Standley, 2008). More specifically, when chanting or singing is included in the classroom, children tend to have improved phonological awareness and literacy skills, suggesting that even informal forms of musical training may enhance literacy skills (Standley and Hughes, 1997; Darrow, 2009; Bolduc and Lefebvre, 2012).

Gordon and colleagues propose that the way forward is to focus on what is transferred from music to language and how it is being transferred. With this goal in mind, they plausibly argue that rather than a direct transfer between music and literacy, improved reading fluency is the gradual result of enhanced auditory discrimination leading to improved rhyming skills that in turn facilitates phonological awareness (see also Besson et al., 2011; Dittinger et al., 2016 for further consideration of this approach). In support, musical training has been shown to positively impact reading skills even when elements of the music and language tasks differ in surface features, cues, and demand characteristics (Gordon et al., 2015). Significant strides may emerge by examining both individual differences in innate musical traits and the underlying neural mechanisms of music and language (e.g., Moreno, 2009; Moreno et al., 2011; François and Schön, 2014). Moreover, it is difficult to ascertain learning outcomes and the effects of learning on brain and behavior when experimental paradigms do not address the complex process of learning. Though pre- and post-tests and group comparisons provide important categorical distinctions of learning, the critical knowledge of what is being transferred and the dynamics of learning (Tuller et al., 2008) must include more detailed longitudinal examination of the points in-between (François et al., 2013; Chobert et al., 2014; Habib et al., 2016). Though hemispheric dominances for music and language are present, overlapping neural substrates and shared cerebral networks indicate that factors such as predispositions to musicianship, exposure to music, and physiological differences all contribute to the development of neural circuits for language (Zatorre, 2001; Koelsch et al., 2002; Brown et al., 2006; Patel, 2011; Schön and François, 2011). As such the ability of musical training paradigms to affect reading skills may be informed by pre-existing abilities, experience, and neural architecture.

In conclusion, continued examination of positive transfer effects from musical training to literacy skills furthers our understanding of the complex connections between music and language, and their shared neurocognitive features. By outlining longitudinal studies that would address study design factors and further the examination of the neural mechanisms underlying musical training driven learning and plasticity, Gordon and colleagues provide us with a compelling example of and future direction toward such research.

## Author contributions

The author confirms being the sole contributor of this work and approved it for publication.

### Conflict of interest statement

The author declares that the research was conducted in the absence of any commercial or financial relationships that could be construed as a potential conflict of interest.
